# Different levels of associations between medical co-morbidities and preterm birth outcomes among racial/ethnic women enrolled in Medicaid 2014–2015: retrospective analysis

**DOI:** 10.1186/s12884-020-2722-8

**Published:** 2020-01-13

**Authors:** Hyewon Lee, Ilya Okunev, Eric Tranby, Michael Monopoli

**Affiliations:** 1 Department of Dentistry, Mount Sinai Hospital, 1 Gustave Place, New York, NY 10029 USA; 2 Analytics and Evaluation, DentaQuest Partnership for Oral Health Advancement, 465 Medford Street, Boston, MA 02129 USA; 3 Data and Impact, Analytics and Evaluation, DentaQuest Partnership for Oral Health Advancement, 465 Medford Street, Boston, MA 02129 USA; 4 Grant Strategy, DentaQuest Partnership for Oral Health Advancement, 465 Medford Street, Boston, MA 02129 USA

**Keywords:** Preterm birth, Racial factors, Disparities, Medicaid

## Abstract

**Background:**

The causes of preterm birth are multi-dimensional, including delayed and inadequate prenatal services as well as other medical and socioeconomic factors. This study aimed to examine the different levels of association between preterm birth and major medical co-morbidities among various racial/ethnic women enrolled in Medicaid.

**Methods:**

This is a retrospective analysis of 457,200 women aged between 15 and 44 with a single live birth from the IBM® MarketScan® Multi-State Medicaid Database from 2014 to 2015. Preterm birth, defined by delivery before 37 completed weeks of gestation, was the primary dependent variable. All births were dichotomously categorized as either preterm or full-term birth using the International Classification of Diseases, Ninth Revision, Clinical Modification codes. Independent variables included race/ethnicity, categorized as non-Hispanic white, non-Hispanic black, Hispanic, or other. Medical co-morbidities included smoking, drug dependence, alcohol dependence, diabetes, and hypertension. Total healthy prenatal visit count and high-risk prenatal visit encounters identified during 30 weeks prior to the delivery date were included in the analysis.

**Results:**

A significantly higher preterm birth rate was found in black women after controlled for medical co-morbidities, age, prenatal visit count, and high-risk pregnancy. Different levels of association between preterm birth outcome and major medical co-morbidities were examined among various racial/ethnic women enrolled in Medicaid. Drug dependence was associated with higher odds of preterm birth in black (OR = 2.56, 95% CI [1.92–3.41]) and white women (OR = 2.12, 95% CI [1.91–2.34]), when controlled for other variables. In Hispanic women, diabetes (OR=1.44, 95% CI [1.27, 1.64]) and hypertension (OR=1.98, 95% CI [1.74, 2.26]) were associated with higher odds of preterm birth. White women diagnosed with drug dependence had a 14.0% predicted probability of preterm birth, whereas black women diagnosed with drug dependence had a predicted probability of preterm birth of 21.5%.

**Conclusions:**

The associations of medical co-morbidities and preterm births varied across racial and ethnic groups of women enrolled in Medicaid. This report calls for future research on racial/ethnic disparity in preterm birth to apply integrative and qualitative approaches to understand the disparity from a contextual perspective, especially for vulnerable pregnant women like Medicaid enrollees.

## Introduction

Preterm birth is defined as the delivery of an infant prior to 37 weeks of gestation and may lead to increased infant mortality and morbidity as well as emotional stress and increased financial burdens to families and to society [1, 2]. Maternal age (both young and advanced at conception), history of smoking, substance abuse, obstetrical history, diabetes, and hypertension are common risk factors for preterm birth [1, 3–6]. The timing of initiation and frequency of prenatal care, often measured by the Kotelchuck or Kessner Index, were found to be significantly associated with preterm birth outcomes [7–9]. Adequate prenatal care can assess a woman’s health and support for recommended care while reducing risky behaviors [10].

Race and ethnicity are also associated with preterm birth; Black women have higher rates of preterm birth than other racial groups [1]. Nationally, the preterm birth rate among black women was 13% in 2014 and 14% in 2016, while white women had a preterm birth rate of 9% both in 2014 and 2016 [3, 11]. As race/ethnicity is interwoven with multiple social, economic, and cultural issues, however, the precise cause of this disparity is not clearly known. Social determinants of health, such as maternal educational level, family income, housing situation, partner support as well as community factors can also play an important role in accounting for these disparities in preterm birth outcomes [1, 12–15].

Enrollment in Medicaid, which covers nearly half of all births in the United States, is itself an independent predictor of preterm delivery [12, 13, 16–19]. Medicaid beneficiaries share common characteristics, such as lower income and health literacy, among other risk factors, which in turn impact their overall health, lifestyle, and access to and utilization of prenatal health services and birth outcomes [12, 13, 16, 18]. The percentage of Medicaid-enrolled pregnant women who received more than 80% of the expected number of prenatal visits ranged from 1 to 85% with considerable geographic variation across states [10, 20]. Thus, given the various risk factors and the consistently higher preterm birth rates among women enrolled in Medicaid, it is critical to examine the interrelationship between these risk factors and preterm birth among different racial and ethnic groups enrolled in Medicaid.

## Methods

The current study is a retrospective analysis of de-identified administrative claims data available from the IBM® MarketScan® Multi-State Medicaid Database, formerly Truven MarketScan® Multi-State Medicaid Database, from January 1, 2014, and September 30, 2015. This dataset contained unique enrollees from thirteen states, but the individual state is not identified within the data. A cohort of 457,200 women was generated who were aged between 15 and 44 and delivered a single live birth at an inpatient hospital facility (Appendix 1). Stillbirths, multiple gestations, and low birth weight without gestation week information were excluded from the analysis.

Preterm birth was the primary dependent variable which was dichotomously categorized as either preterm or full-term using the International Classification of Diseases, Ninth Revision, Clinical Modification (ICD-9-CM) codes (Appendix 1). In this study, preterm birth included all delivery before 37 completed weeks of gestation, including extremely preterm birth, delivery prior to 28 weeks of pregnancy [21]. Independent variables included race and ethnicity, categorized as non-Hispanic white, non-Hispanic black, Hispanic, or other. Major medical co-morbidities diagnosed at the time of delivery included smoking, drug dependence, alcohol dependence, diabetes, and hypertension, which included both pre-eclampsia and eclampsia, and their ICD-9-CM codes are listed in Appendix 2. Total healthy prenatal visit count and any high-risk prenatal visit encounters identified during 30 weeks prior to the delivery date were included in the analysis using ICD-9-CM codes. Utilizing the prenatal visit frequency recommendations by the American College of Obstetricians and Gynecologists, women were divided into three groups for analysis: women who had no prenatal visit found in the administrative record, those with one to seven visits, and those with eight to 30 visits [20]. Any woman with 31 or more prenatal visits was considered outliers (*n*=56) and therefore excluded from the regression analysis. Any women with at least one high-risk prenatal visit were then categorized as a high-risk pregnancy. In this study, we focused on the mother’s access and utilization of prenatal care services rather than the actual clinical examination or intervention delivered during the prenatal visit. The ICD-9 codes for prenatal visits for normal and high-risk pregnancy are listed in Appendix 3.

Logistic regression analysis was used to estimate rates of preterm birth associated with other modifying factors: age, race, medical co-morbidities, prenatal visit count, and high-risk pregnancy. All analyses were performed in Stata 15 (StataCorp LLC).

## Results

### Preterm birth

**Fig. 1:**
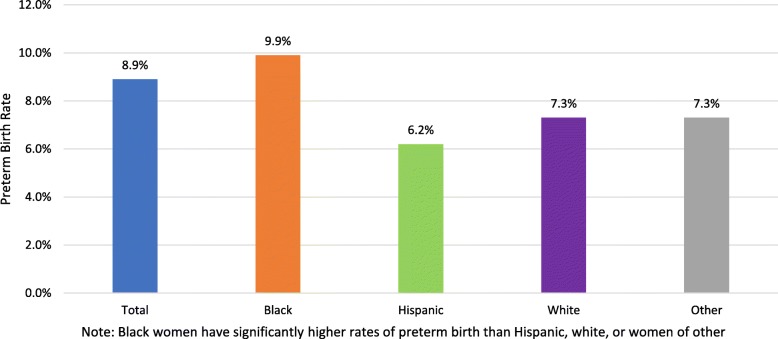
Preterm birth rate by race/ethnicity among women enrolled in Medicaid. ^1^Significantly different from white, Hispanic, and other racial/ethnic women

Among the 457,200 women aged 15 to 44 who gave birth between January 2014 and September 2015, there were 231,942 white, 141,392 black, 30,903 Hispanic, and 52,963 were of another race or ethnicity. The preterm birth rate within the overall cohort was 8.9%. This was highest for black women (9.9%) compared to both white (7.3%) and Hispanic women (6.2%) (Fig. 1).

### Perinatal service utilization

**Fig. 2:**
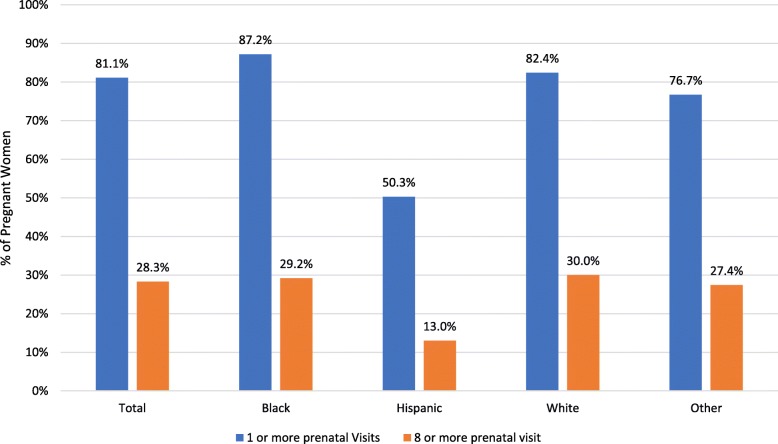
Proportion of women enrolled in Medicaid who accessed prenatal visit during 30 weeks prior to delivery and women who accessed at least one postnatal visit during 12 weeks after delivery

More than four in five women (81.1%) accessed at least one prenatal visit during 30 weeks prior to delivery (Fig. 2). Hispanic women had the lowest prenatal visit rate (50.3%) compared to black (87.2%), white (82.43%), and other racial/ethnic women (76.7%). Less than one thirds (28.3%) of women included in this study had 8 or more prenatal visits 30 weeks prior to birth. Approximately one third (32.2%) had at least one high-risk pregnancy visit during 30 weeks prior to delivery.

### Medical co-morbidities at the time of delivery associated with preterm birth

The prevalence of hypertension was the highest for black women (16.1%) (Table 1). Hispanic women had the highest prevalence of diabetes among all groups (11.7%), and white women had a significantly higher prevalence of drug dependence (1.3%) and smoking (0.5%) than both black and Hispanic women.

**Table 1:** Major medical co-morbidity rates in pregnant women enrolled in Medicaid

	Smoking	Drug Dependence	Diabetes	Alcohol Dependence	Hypertension
Black	353 (0.25%)	254 (0.18%)	9048 (6.40%)	226 (0.16%)	22,747 (16.09%)
Hispanic	15 (0.05%)	28 (0.09%)	3622 (11.72%)	25 (0.08%)	2627 (8.50%)
White	1252 (0.54%)	2968 (1.28%)	16,744 (7.22%)	348 (0.15%)	25,649 (11.06%)
Other	169 (0.32%)	397 (0.75%)	4639 (8.76%)	79 (0.15%)	5640 (10.65%)
Total	1789 (0.39%)	3647 (0.80%)	34,053 (7.45%)	678 (0.15%)	56,663 (12.39%)

### Multivariate analysis

In the regression analysis, the odds of preterm birth among black women was 35% higher than those for white women, controlling for age, smoking, drug and alcohol dependence, diabetes, hypertension, high-risk pregnancy, and the total number of prenatal visits occurring 30 weeks prior to delivery (OR=1.35; 95% CI [1.32, 1.38]) (Table 2). The odds of preterm birth in Hispanic women were 17% less than white women after adjusting for all other variables (OR=0.83; 95% CI [0.76, 0.87]). The odds of preterm birth were more than double for women diagnosed with drug dependence (OR = 2.21; 95% CI [2.02, 2.42]) or alcohol dependence (OR = 2.09; 95% CI [1.71, 2.55]) after adjusting for other variables. Hypertension (OR=1.82, 95% CI [1.77, 1.87]), maternal smoking (OR=1.50) and diabetes (OR=1.27) were also associated with higher odds of preterm birth.

**Table 2:** Logistic Regression Model Estimating Preterm Rates among Women Enrolled in Medicaid

Logistic Regression Model Estimating Preterm Birth among Women Enrolled in Medicaid												
	All Women			White Women			Black Women			Hispanic Women		
Independent Variable	Odds Ratio		95% C.I.	Odds Ratio		95% C.I.	Odds Ratio		95% C.I.	Odds Ratio		95% C.I.
Age												
Age	1.00		1.00–1.00e	1.00		1.00–1.00e	1.00		1.00–1.00e	1.00		0.99–1.01
Race												
(Reference: White)	–		–	–		–	–		–	–		–
Black	1.35	**	1.32–1.38	–		–	–		–	–		–
Hispanic	0.83	**	0.79–0.87	–		–	–		–	–		–
Other	0.99		0.95–1.02	–		–	–		–	–		–
Medical Conditions During Pregnancy												
Smoking	1.50	**	1.3–1.73	1.64	**	1.38–1.94	1.07		0.77–1.48	2.03		0.45–9.11
Drug Dependence	2.21	**	2.02–2.42	2.12	**	1.91–2.34	2.56	**	1.92–3.41	1.42		0.42–4.78
Alcohol Dependence	2.09	**	1.71–2.55	2.11	**	1.58–2.8	2.03	**	1.47–2.81	1.45		0.34–6.29
Diabetes	1.27	**	1.23–1.32	1.27	**	1.2–1.34	1.25	**	1.17–1.33	1.44	**	1.27–1.64
Hypertension	1.82	**	1.77–1.87	1.77	**	1.7–1.85	1.83	**	1.76–1.91	1.98	**	1.74–2.26
Prenatal Visits												
(Reference: No High Risk Prenatal Encounter)	–		–	–		–	–		–	–		–
1+ High Risk Prenatal Encounter	1.39	**	1.35–1.42	1.45	**	1.4–1.5	1.28	**	1.24–1.33	1.55	**	1.38–1.74
(Reference: 8+ Prenatal Visits)	–		–	–		–	–		–	–		–
No Prenatal Visits	1.78	**	1.72–1.84	1.63	**	1.55–1.72	2.04	**	1.92–2.17	1.58	**	1.34–1.87
Between 1 and 7 Prenatal Visits	1.68	**	1.64–1.73	1.58	**	1.52–1.64	1.83	**	1.75–1.91	1.47	**	1.24–1.74
Constant	0.04	**	0.04–0.04	0.04	**	0.04–0.05	0.05	**	0.05–0.06	0.04	**	0.03–0.05
Number of Observations	457,144			231,912			141,374			30,902		
Model Chi-Square Test	6258.3	**	(12 *df)*	2338.4	**	(9 *df)*	2154.6	**	(9 *df)*	233.6	**	(9 *df)*
* *p* < .01; **p < .001.												

When stratified, drug dependence was associated with higher odds of preterm birth in both black and white women after adjusting for age, prenatal visit count and other medical conditions. The level of the association was higher in black women (OR = 2.56, 95% CI [1.92, 3.41]) compared to that of white women (OR = 2.12, 95% CI [1.91, 2.34]) (Table 2). Neither drug nor alcohol dependence was significantly associated with higher odds of preterm birth in Hispanic women (*p*> 0.05). Maternal smoking was associated with higher odds of preterm birth only for white women (OR=1.64, 95% CI [1.38, 1.94]). In Hispanic women, diabetes (OR=1.44, 95% CI [1.27, 1.64]) and hypertension (OR=1.98, 95% CI [1.74, 2.26]) were associated with higher odds of preterm birth (Table 2). Maternal smoking was associated with higher odds of preterm birth only for white women (OR=1.64, 95% CI [1.38, 1.94]).

**Fig. 3:**
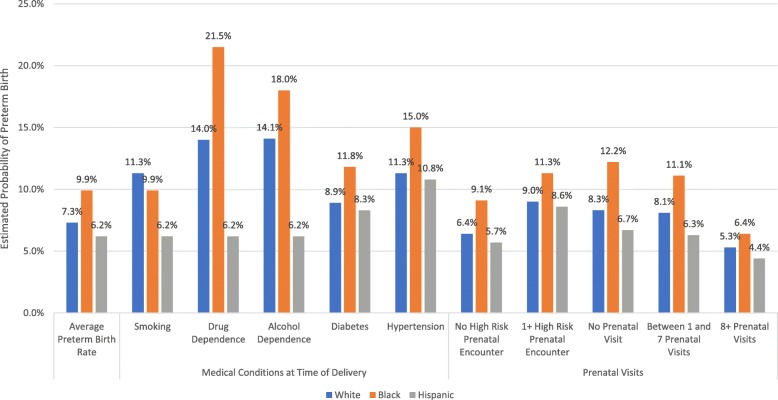
Estimated probability of preterm birth among women of different races/ethnicity, by medical co-morbidities at the time of delivery, and prenatal visits

The predicted probabilities of preterm birth among women of different races and ethnicities were estimated by medical co-morbidities and by total prenatal visit counts prior to delivery (Fig. 3). White women diagnosed with drug dependence had a 14.0% predicted probability of preterm birth, whereas black women with drug dependence had a predicted probability of preterm birth of 21.5%.

For all racial and ethnic women, those with eight or more prenatal visits were associated with a decreased predicted probability of preterm birth. Compared with women who did not have any prenatal visit, the predicted probability of preterm birth among women with 8 or more prenatal visits decreased from 12.2 to 6.4% in black women, 8.3 to 5.3% in white women, and from 6.7 to 4.4% in Hispanic women, adjusted for age and other medical co-morbidities.

## Discussion

Among 457,200 Medicaid-enrolled women from the IBM/Watson Truven Multi-State Medicaid core dataset, black women had 35% higher odds of preterm birth than white women, even when controlling for age, prenatal visit number, and major medical co-morbidities, including smoking, drug dependence, alcohol dependence, diabetes, and hypertension. While a higher preterm birth rate in black women is a well-known finding, this study showed that racial/ethnic disparity in preterm birth persists among women enrolled in Medicaid, who are expected to share some of the common characteristics: significant financial hardship, less likely to be married, and more medical co-morbidities [7, 18, 19].

When stratified by race and ethnicity, each medical condition affected preterm birth outcomes differently. Most notably, black and white women showed strong associations between drug or alcohol dependence and preterm birth outcomes. On the other hand, Hispanic women showed the highest odds of preterm birth associated with diabetes and hypertension, which are more related to lifestyle rather than risky behaviors.

The study also provided continued evidence of the Hispanic Paradox, as observed in previous studies: Hispanic women had the lowest utilization of prenatal visits, but they had the lowest preterm birth outcomes among all racial/ethnic groups in the study. Future studies that evaluate Hispanic women’s prenatal health status and how their unique social and cultural context influence health behaviors are necessary [3, 22].

The study finding demands a better understanding of the underlying influences of various medical conditions and its associated social factors to different groups of women. Until now, it is still unclear how race and ethnicity play their role as a predominant determinant of preterm birth. Birth outcomes are different among Hispanic women with different length of immigration years [23]. Risk behaviors like drug dependence and chronic medical conditions such as diabetes and hypertension often coincide with other medical and socioeconomic vulnerabilities and cultural contexts [1, 8]. Contextual factors, such as distressed neighborhood, thought to explain up to two-thirds of racial disparity in the preterm birth rate among black and white women [8, 24, 25]. In future research, different profiles of pregnant women can be introduced beyond self-reported racial/ethnic classification that may account for these contextual factors.

The study also showed adequate prenatal care visits were strongly associated with preterm birth outcomes in all racial/ethnic groups. There have been diverse approaches to support pregnant women in accessing early and adequate prenatal care and reduce preterm birth outcomes. A multi-state study found that merely increasing obstetric providers was not associated with increased utilization of office-based prenatal care by black Medicaid enrolled pregnant women [8, 26]. A recent Cochrane review concluded that midwife-led continuity models of care showed clear evidence of reducing preterm birth and perinatal death [22]. These findings again highlight the need to understand how risk-specific and community-engaging models of prenatal care could be implemented in different profiles of pregnant women to prevent preterm birth, especially in Medicaid populations.

The analysis was performed on the IBM® MarketScan® Multi-State Medicaid Database of nearly a half-million Medicaid enrolled women from 13 states. The dataset does not identify individual states, and we cannot examine the trends identified in this study are the result of geography and state-level characteristics regarding Medicaid policies. While the vast number of subjects increases the power of the study, we may not be able to generalize the study outcome to other states or non-Medicaid populations as the dataset is not a nationally representative sample. In addition, the dataset did not provide socioeconomic information such as marital status, employment status, or education level. We may assume that women enrolled in Medicaid are likely to share certain sets of socioeconomic characteristics, such as low-income status [12, 13, 18, 19]. However, future research should include ways to categorize pregnant women enrolled in Medicaid, considering risky behaviors, daily lifestyle, and social support elements.

## Conclusions

The associations of medical co-morbidities and preterm births varied across racial and ethnic groups enrolled in Medicaid. This report calls for future research on racial/ethnic disparity in preterm birth to apply more integrative and qualitative approaches to ultimately understand the disparity from a contextual perspective. This comprehensive understanding will help identify prenatal care strategies and policies to reduce preterm birth outcomes among various profiles of pregnant women, especially the ones from vulnerable backgrounds, such as Medicaid enrollees.

## Data Availability

The data that support the findings of this study are available from IBM/Watson Truven Multi-State Medicaid core dataset, but restrictions apply to the availability of these data, which were used under license for the current study, and so are not publicly available. Data are however available from the authors upon reasonable request and with permission of IBM/Watson Truven.

## Appendix 1

**Table 3:** Single liveborn birth event defined by ICD-9-CM codes

Diagnosis	ICD-9-CM
Pre-term birth	
Early onset of delivery, unspecified as to episode f care or not applicable	644.2X
Disorders relating to other preterm infants	765.1X
Weeks of gestation (born prior to 37 week of gestation)	765.20–765.28
Full-term birth (Including low-birth weight diagnosis without preterm birth diagnosis and post-term delivery)	765.0X
	645.11
	650
	669.7X
	V27.0
	V30.00
	V30.01

## Appendix 2

**Table 4:** Medical Co-morbidity by ICD-CM9/10 codes

Diagnosis at birth	ICD-CM 9
Smoking	305.1
Hypertension/Eclampsia	642.XX
Drug Dependence	292.0
	304.XX
Alcohol Dependence	303.XX
	305.XX
Diabetes	648.0X
	648.8X

## Appendix 3

**Table 5:** Prenatal and postnatal visit (delete post-natal visit)

Diagnosis	ICD-CM 9
Healthy prenatal visit	V22.0
	V22.1
	V22.2
High risk prenatal visit	V23.XX

## Abbreviations

IBM: International Business Machines
ICD-9-CM: International Classification of Diseases, Ninth Revision, Clinical Modification (ICD-9-CM) codes
